# The accuracy of radiographic peer review in identifying primary hip and knee arthroplasties at higher risk of early aseptic revision: an observational cohort study from the JointCare registry of South Africa

**DOI:** 10.2340/17453674.2026.46431

**Published:** 2026-09-03

**Authors:** Antoine S D DYMOND, Ian D LEARMONTH, Ponky FIRER, Aashish DIAYAR

**Affiliations:** 1 JointCare, Sandton, Gauteng, South Africa; 2 University of Bristol, Bristol, UK; 3 Linksfield Orthopaedic Sports and Rehabilitation Centre, Johannesburg, Gauteng, South Africa

## Abstract

**Background and purpose:**

Healthcare value might be improved with radiographic peer review to evaluate surgical quality. While numerous radiographic recommendations are available, peer review differs by including expert appraisal. We aimed to assess the predictive value of radiographic peer review in identifying primary hip and knee arthroplasties at higher risk of aseptic revision.

**Methods:**

A retrospective observational cohort study was conducted to compare aseptic revision rates with peer review scores, using data from the JointCare registry of South Africa. Matching and weighting was applied among cohorts, and revision rates were compared using Cox proportional hazards regression.

**Results:**

The sample consisted of 7,218 arthroplasties performed between January 2020 and June 2023. 5,126 arthroplasties were scored as “good,” 1,931 as “acceptable,” and 161 as “suboptimal.” “Suboptimal” arthroplasties had a significantly higher hazard ratio (HR) for aseptic revision than “good” arthroplasties (HR 9.4, 95% confidence interval [CI] 3.6–24.5). Furthermore, “suboptimal” total hip (HR 11.4, CI 2.6–49.7), total knee (HR 5.7, CI 1.3–25.6), and medial unicompartmental knee arthroplasties (HR 9.8, CI 2.0–47.4) had significantly higher HRs than “good” ones. Overall and for each procedure, “acceptable” arthroplasties had higher HRs than “good” arthroplasties; however, these results were not statistically significant.

**Conclusion:**

Radiographic review by experienced arthroplasty surgeons was able to identify arthroplasties at higher risk of aseptic revision. As such, radiographic review could be used for quality improvement by identifying directions for surgical improvement.

Economic constraints, and consequent austerity measures, profoundly impact healthcare globally. Healthcare purchasers are entitled to seek value for cost-effective treatment, where value equals outcome quality divided by cost [1]. Radiographic peer review is a potential avenue for improving value through the identification of surgical shortcomings.

Postoperative radiographic review is routinely performed to assess hip and knee arthroplasties [2]. Radiographic-analysis applications include training surgeons, a means for a practising physician to critique their or their colleagues’ work, informing expert opinion in legal matters, and, more recently, to assess surgical quality in a national arthroplasty network in South Africa. Recent registry evidence suggests that sensitive radiographic surveillance may complement revision-based monitoring [3]. It may therefore follow that reviewing failures or potential failures may improve future operative quality and, consequently, patient outcomes. How valuable radiographic review is, and how significant a “bad-looking radiograph” is, are key questions.

Numerous radiographic recommendations have been published, each providing various findings ranging from cup inclination and anteversion safe zones [4], to the acceptable degree of femoral notching [5]. Peer review differs, however, as it includes expert appraisal. Healthcare funders and other stakeholders may wish to encourage peer review, subject to cost-vs-benefit analyses, and surgeons may consider joining peer review groups or programs that have been shown to be effective in identifying poorly performed arthroplasties.

Our study explores how accurately JointCare’s radiographic peer review program can identify arthroplasties at a higher risk of aseptic revision. Radiographic review cannot tell if an arthroplasty is definitely going to fail, as there are too many unknowns and stochastic variables for that. The reviewer should be assessing whether the manner in which the arthroplasty has been done has increased the risk of failure. Registry data is used to compare peer review scores with the incidence of early aseptic revision, thus assessing the predictive value of the peer review process.

## Methods

### Study design

A retrospective cohort study from the JointCare registry of South Africa, including data on primary arthroplasties performed between January 2020 and June 2023, was performed. The study is reported according to STROBE guidelines [6].

### Setting and data sources

#### Registry data

Included procedures were performed by 136 surgeons in 71 private hospitals located in 8 of the 9 provinces of South Africa. Hospital-level characteristics and minimum hospital-volume thresholds were not modelled, as the aim was to evaluate peer-review predictive validity across the registry population rather than within hospitals.

The data source was the JointCare registry, which conducts patient follow-up to determine whether a revision took place. JointCare is an optional managed care organization for primary hip and knee arthroplasties in the private sector of South Africa. As such, its registry contains details of primary arthroplasties only, with revision outcomes captured only as an endpoint of these arthroplasties. JointCare’s registry contains data for approximately 14% of the private sector of South Africa [7, Personal communication, Dymond A et al. 2026].

Arthroplasties performed on JointCare’s network are all standard, primary, and unilateral. Arthroplasties where the patient has challenging comorbid diseases, such as renal failure and transplant cases, are not included in the JointCare network.

#### Peer review program

The peer review scores were taken from JointCare’s peer review program. This program has previously been validated through JointCare’s internal analysis and results have been presented at local and international orthopaedic congresses. This is the first publication reporting on the program.

Each arthroplasty in this study underwent radiographic review by at least 1 of 13 reviewers, all of whom were experienced arthroplasty surgeons. Reviewer selection is vital for the accuracy of the peer review program. As such, candidate reviewers are carefully selected. JointCare-partnered surgeons are asked about their interest in being a reviewer annually. Surgeons identified through this process, and other interested surgeons, are then screened. While no specific rules for reviewer selection exist, factors that are taken into consideration include surgical and academic experience in arthroplasty, and concordance of scores with those of existing reviewers. JointCare aims to ensure that the review panel is representative of the surgical community, including representation from different academic institutions, alignment philosophies, use of technology such as robots and navigation, and other relevant clinical perspectives.

The aim of JointCare’s peer review program is to gauge the quality of surgery, and to engage with surgeons on improving future care. Given this context of quality improvement, the review scores were “good” (little room for practical improvement), “acceptable” (some room for improvement), and “suboptimal” (significant room for improvement).

The review process was double-blinded, with patients’, operating surgeons’, and reviewers’ identities being anonymized. The data available to reviewers is outlined in Table 1. Furthermore, statistical analysis was done so that patient-specific and radiographic metric outliers [8] could be marked for the reviewers’ attention.

**Table 1:** Data presented on the hip and knee arthroplasty review forms

Information type Details
Patient metrics
Age, sex, BMI, ASA grade ^**a**^, and primary and comorbid ICD-10 codes ^**a**^
Medical team reports
EMRs from the surgeon, assistant surgeon, anesthetist and physiotherapist. Each EMR is role- and procedure-specific, with most allowing the provider to leave a comment. Comments allow the medical team to provide any additional information they believe the reviewer should know about
Prosthesis classification
Implant name, manufacturer, link to surgical techniques, ODEP rating, bearing material, fixation method, and procedure-specific attributes: head diameter and cup outer-diameter for total hip arthroplasties, and stabilization method for total knee arthroplasties
Hospital metrics
Theatre time, length of hospital admission, total cost, and pharmacy cost. These metrics are each provided with a statistical comparison with other arthroplasties of the same type
Radiographs
* Pre- and postoperative:* The radiographs inform the reviewer on the placement of the implant and provide evidence of the pathology
* Hip radiograph views:* (i) preoperative AP pelvis, (ii) preoperative AP pelvis showing proximal femur ^**b**^ and/or lateral view of hip ^**b**^, (iii) operative planning ^**b**^, (iv) postoperative AP pelvis, and (v) postoperative AP pelvis showing proximal femur ^**b**^ and/or lateral view of hip ^**b**^
* Knee radiograph views:* (i) preoperative AP standing, (ii) preoperative lateral, (iii) preoperative Rosenberg ^**b**^, (iv) preoperative AP stress ^**b**^, (v) preoperative skyline patella ^**b**^, (vi) preoperative long-leg (hip–knee–ankle) AP standing ^**b**^, (vii) postoperative AP straight leg, (viii) postoperative lateral, (ix) postoperative skyline patella ^**b**^, and (x) postoperative long-leg (hip–knee–ankle) AP standing ^**b**^
Postoperative radiographs were those obtained during routine early follow-up, generally within 6 weeks of surgery, although the exact timing varied according to the treating surgeon’s pathway
Radiographic metrics ^**c**^
Tabulated together with the results from a statistical analysis. Outliers in the lower and upper 10% are highlighted for the reviewers’ attention. Histograms can also be accessed should the reviewer wish to view the statistical distribution for a metric
Intraoperative media
May also be included if the medical team wishes to upload it. Media may either be images or videos, with the media often being captured on a smartphone or similar device

a Only captured for primary arthroplasties performed in or after 2022.

b Optional radiographic views.

c For total hip arthroplasties: leg length discrepancy, medialization, proximalization, cup inclination, anteversion, and penetration of ilioischial line. For total knee arthroplasties: medial proximal tibial angle, lateral distal femoral angle, posterior tibial slant, notching distance, and hip–knee–ankle angles. For medial unicompartmental knee arthroplasties: varus rotation, flexion–extension angle, and the posterior overhang of the femoral component; and varus angle, posteroinferior angle, medial overhang, anterior overhang, and posterior overhang of the tibial component, if measured. If the radiograph required for the radiographic metric is missing, then that metric is left off the review form, as is often the case for hip–knee–ankle measurements. Refer to the annual reports from the JointCare Registry, available at https://joint-care.co.za/ for further details on the radiographic metrics captured including completeness rates.

AP = anterior-to-posterior; ASA = American Society of Anesthesiologists; BMI = body mass index; EMR = electronic medical record; ODEP = Orthopaedic Data Evaluation Panel.

Hard rules or prespecified cut-offs that automatically generated the overall review score were intentionally not used, because published radiographic recommendations require contextual interpretation and may vary by implant, procedure type, and clinical circumstance. Reviewers were, therefore, asked to integrate the structured radiographic findings with their arthroplasty experience when assigning the overall score. In this program, the purpose of the score was to judge whether the observable technical features suggested little, some, or significant room for improvement, based on reviewers’ opinions, which were guided by both evidence and their own clinical experience.

Excessive administrative load is likely to create resistance, and may contribute to surgeon burnout [9,10]. Focus was, therefore, directed on minimizing administration, particularly for the medical team. Routine data-capture aspects like uploading and measuring radiographs were outsourced. The financial and peer review systems were also integrated so that data available from sources such as the prosthesis and hospital bills could be used without having to be recaptured. This ease of use greatly enhanced the willingness of medical teams to participate in the peer review program.

Reviewers were asked about implant familiarity, including separate acetabular and femoral-system familiarity for total hip arthroplasties, because positioning recommendations vary by implant. Further questions addressed implant positioning, sizing, and alignment. They were then asked what they would have done differently, and to provide an overall score (“good,” “acceptable,” or “suboptimal”) for the arthroplasty. They could also provide additional comments should they wish. This overall score reflected the reviewer’s synthesis of the case rather than a simple count of abnormal radiographic features.

If “suboptimal” was selected as the operation score, 2 additional reviewers were assigned. These 3 reviews were then discussed at a reviewers’ meeting, after which a final consensus score was given.

The cohorts compared were defined using each arthroplasty’s overall score.

### Participants

#### Inclusion and exclusion criteria

Patients who underwent total hip, total knee, and medial unicompartmental knee arthroplasties on JointCare’s network during the defined study period were included.

Arthroplasties with mortality within 1 year of surgery were excluded to reduce confounding and other biases. Patients diagnosed with osteonecrosis and rheumatoid arthritis were excluded due to their low volume and differing base revision risk. Cases that were revised due to non-radiographically predictable indications were also excluded, as they are outside the scope of this study. Periprosthetic fractures were included when they resulted in aseptic revision. They were otherwise excluded, as non-revision fracture events and non-revision reoperations were not part of the primary endpoint for this study. To reduce a potential information bias, aseptic revisions were excluded where information that a revision occurred was available to the radiographic reviewers.

#### Pre-matching cohort characteristics

Cohort covariate compositions differed significantly, as summarized in Table 2. The procedure type composition varied significantly across the cohorts (P < 0.001). The difference in medial unicompartmental knee arthroplasty composition of 7.4% between the good-review-score and the suboptimal-review-score cohorts was expected to be relevant. Specifically given that revision rates differ between total hip, total knee, and medial unicompartmental knee arthroplasties, the different procedure compositions were hypothesized to result in some confounding. Although several baseline comparisons were statistically significant before matching, many absolute differences were small, which is unsurprising in a cohort of this size. Accordingly, the post-matching balance diagnostics shown in Figure 2 and Table 3 are more relevant to the interpretation of the main analyses than the unadjusted, baseline P values alone.

**Table 2:** Demographics and procedure-related factors for cohorts. Values are count (%)

Variable	Review score			P value
	Good (n = 5,126)	Acceptable (n = 1,931)	Suboptimal (n = 161)	
Procedure				< 0.001 ^**a**^
THA	1,971 (38)	969 (50)	71 (44)	
TKA	2,870 (56)	830 (43)	69 (43)	
mUKA	285 (5.6)	132 (6.9)	21 (13)	
Age, median	68	68	67	0.02 ^**b**^
IQR	61–75	60–74	60–73	
Sex				0.05 ^**a**^
Female	3,119 (61)	1,212 (63)	111 (69)	
Male	2,007 (39)	719 (37)	50 (31)	
BMI, median	29.7	29.9	29.8	0.3 ^**b**^
IQR	26.0–34.4	26.1–34.7	26.3–35.3	
ASA grade (n = 4,880)				> 0.9 ^**a**^
1	818 (23)	274 (22)	26 (24)	
2	2,520 (71)	880 (72)	78 (71)	
3	209 (5.9)	64 (5.3)	6 (5.5)	
4	4 (0.1)	1 (0.1)	0 (0.0)	
Year of surgery				< 0.001 ^**a**^
2020	640 (12)	333 (17)	26 (16)	
2021	945 (18)	382 (20)	26 (16)	
2022	2,124 (41)	761 (39)	63 (39)	
2023	1,417 (28)	455 (24)	46 (29)	
Implant ODEP rating				< 0.001 ^**a**^
Not rated	1,653 (32)	510 (27)	43 (27)	
Rated	3,473 (68)	1,421 (73)	118 (73)	
Mode of implant fixation				< 0.001 ^**a**^
Cemented	2,208 (43)	664 (34)	60 (37)	
Uncemented	2,222 (43)	1,042 (54)	86 (53)	
Hybrid	689 (13)	221 (11)	14 (8.7)	
Reverse-hybrid	7 (0.1)	4 (0.2)	1 (0.6)	

a Chi-square test.

b Kruskal–Wallis test.

ASA = American Society of Anesthesiologists; BMI = body mass index; IQR = interquartile range; mUKA = medial unicompartmental knee arthroplasty, ODEP = Orthopaedic Data Evaluation Panel, THA = total hip arthroplasty, TKA = total knee arthroplasty.

**Table 3:** Post-matching demographics and procedure-related factors for cohorts. Values are effective sample sizes (ESS) and (%)

Variable	Review score			P value
	Good (ESS = 5,034)	Acceptable (ESS = 1,687)	Suboptimal (ESS = 112)	
Procedure				0.9 ^**a**^
THA	2,097 (42)	707 (42)	49 (44)	
TKA	2,633 (52)	878 (52)	56 (50)	
mUKA	304 (6.0)	102 (6.0)	7 (6.3)	
Age, median	68	68	67	0.7 ^**b**^
IQR	61–74	61–74	60–74	
Sex				0.1 ^**a**^
Female	3,067 (61)	1,061 (63)	77 (68)	
Male	1,967 (39)	626 (37)	35 (32)	
BMI, median	29.7	29.7	29.8	0.2 ^**b**^
IQR	26.0–34.4	26.1–34.7	26.6–35.4	
ASA grade (ESS = 4,624)			0.9 ^**a**^	
1	808 (23)	236 (22)	15 (21)	
2	2,475 (71)	766 (72)	54 (73)	
3	204 (5.8)	55 (5.2)	5 (6.5)	
4	4 (0.1)	1 (0.1)	0 (0.0)	
Year of surgery				> 0.9 ^**a**^
2020	689 (14)	239 (14)	16 (14)	
2021	944 (19)	316 (19)	22 (20)	
2022	2,059 (41)	688 (41)	44 (39)	
2023	1,342 (27)	444 (26)	30 (27)	
Implant ODEP rating				0.1 ^**a**^
Not rated	1,542 (31)	520 (31)	27 (24)	
Rated	3,492 (69)	1,167 (69)	85 (76)	
Mode of implant fixation				> 0.9 ^**a**^
Cemented	2,049 (41)	685 (41)	45 (40)	
Uncemented	2,335 (46)	782 (46)	52 (47)	
Hybrid	642 (13)	217 (13)	15 (14)	
Reverse-hybrid	8 (0.2)	3 (0.2)	0 (0.2)	

a Rao–Scott F-test.

b Kruskal–Wallis test.

For abbreviations, see Table 2.

### Variables

The outcome variable of interest was the aseptic revision rate. Revision rates are affected by various factors, of which only some are discernible via radiographic review, such as implant placement and some aspects of patient selection. These radiographic variables were the predictors accounted for in the peer review scores used to define the cohorts.

Confounders with known values included: the type of arthroplasty being performed, the primary ICD-10 code, patient age, patient sex, patient body mass index (BMI), and prosthesis type. The American Society of Anesthesiologists (ASA) Physical Status was known for 68% of arthroplasties. Confounders with unknown values included the patient’s activity profile, psychology, income level, race, culture, and sex association [11].

### Outcome

Aseptic revisions were defined as any operation where 1 or more components are added to, removed from, or modified in a joint replacement, for an indication unrelated to infection [12]. Surgical procedures following arthroplasty not meeting this definition are defined as reoperations in JointCare’s registry, and were not analyzed in this study. Examples of procedures defined as reoperations, rather than revisions, include open reduction and internal fixation of periprosthetic fractures not involving the insertion, removal, or modification of any primary arthroplasty component, and dislocations requiring open reduction without insertion, removal, or modification of any primary arthroplasty component. Dislocations requiring closed reduction without revision or reoperation are not captured as outcomes in the JointCare registry. Where possible, the surgeon who performed the revision or reoperation was contacted to confirm the classification of and indication for the surgical procedure performed. The diagnosis of aseptic loosening is determined by the surgeon’s individual diagnostic method rather than through standardized diagnostic criteria.

Revision survivorship data was obtained by JointCare through annual patient or family follow-up. Revision, reoperation, and death events, as well as reports that none had occurred, were captured. Follow-up ended when the patient was revised, died, opted out, or became uncontactable. Early complications were also captured during routine surgical follow-up, generally within 6 weeks. The data for all other variables came from the JointCare network and associated peer review program as is shown in Table 1. No follow-up methods specific to this study were employed, as all the revision data was already captured in the JointCare registry.

### Statistics

A matching-and-weighting time-to-event approach was used to improve comparability and reduce confounding given the differing baseline characteristics before analysis (Table 2). The matching procedure used age, sex, BMI, year of surgery, procedure type, the Orthopaedic Data Evaluation Panel (ODEP) rating, and fixation method. This was performed in R (v4.5.2) using the MatchIt (v4.7.2), quickmatch (v0.2.3) and cobalt (v4.6.1) packages (R Foundation for Statistical Computing, Vienna, Austria). For procedure-specific subgroup analyses, the same approach was used but procedure type itself was omitted. Matching was performed using generalized full matching on a robust Mahalanobis distance matrix with reference-invariant contrasts, after which average-treatment-effect (ATE)-targeted matching weights were derived. Thus, cases were not directly matched on the raw covariates themselves. Covariate balance after matching was assessed using absolute standardized mean differences (ASMDs), design-based Kruskal–Wallis tests, and Rao–Scott F-tests.

The primary outcome model was a weighted Cox proportional hazards model for time to aseptic revision, fitted using the ATE-derived matching weights with the R survival (v3.8.3) and survminer (v0.5.1) packages. Sandwich-robust standard errors and Efron’s method for ties were used. To provide doubly robust estimation, any covariates that remained imbalanced after matching (ASMD ≥ 0.10 and/or post-matching design-based P ≤ 0.05) were added to the weighted Cox model as additional covariates. The good-review-score cohort served as the reference category for hazard-ratio estimation. Proportional hazards assumptions were assessed using Schoenfeld residual tests and graphical checks. Because competing-risk methods were not used, the plotted curves are weighted Kaplan–Meier estimates rather than competing-risk cumulative-incidence functions.

All statistical tests were 2-tailed and conducted at a Type I error rate of 5% (α = 0.05). Results are reported with both 95% confidence intervals (CIs) and P values.

### Ethics, registration, data sharing plan, funding, use of AI, and disclosures

This study was approved by the Foundation for Professional Development Research Ethics Committee (06/2025). A waiver for informed consent was granted in accordance with the South African guidelines for ethics in research.

The code used for statistical analyses is provided as supplementary material. The anonymized dataset may be provided on reasonable request to the corresponding author.

This study received no external funding. JointCare provided access to registry and peer-review data.

No AI tools were used for the purpose of this study.

Complete disclosure of interest forms according to ICMJE are available on the article page, doi: 10.2340/17453674.2026.46431

## Results

**Figure 1:**
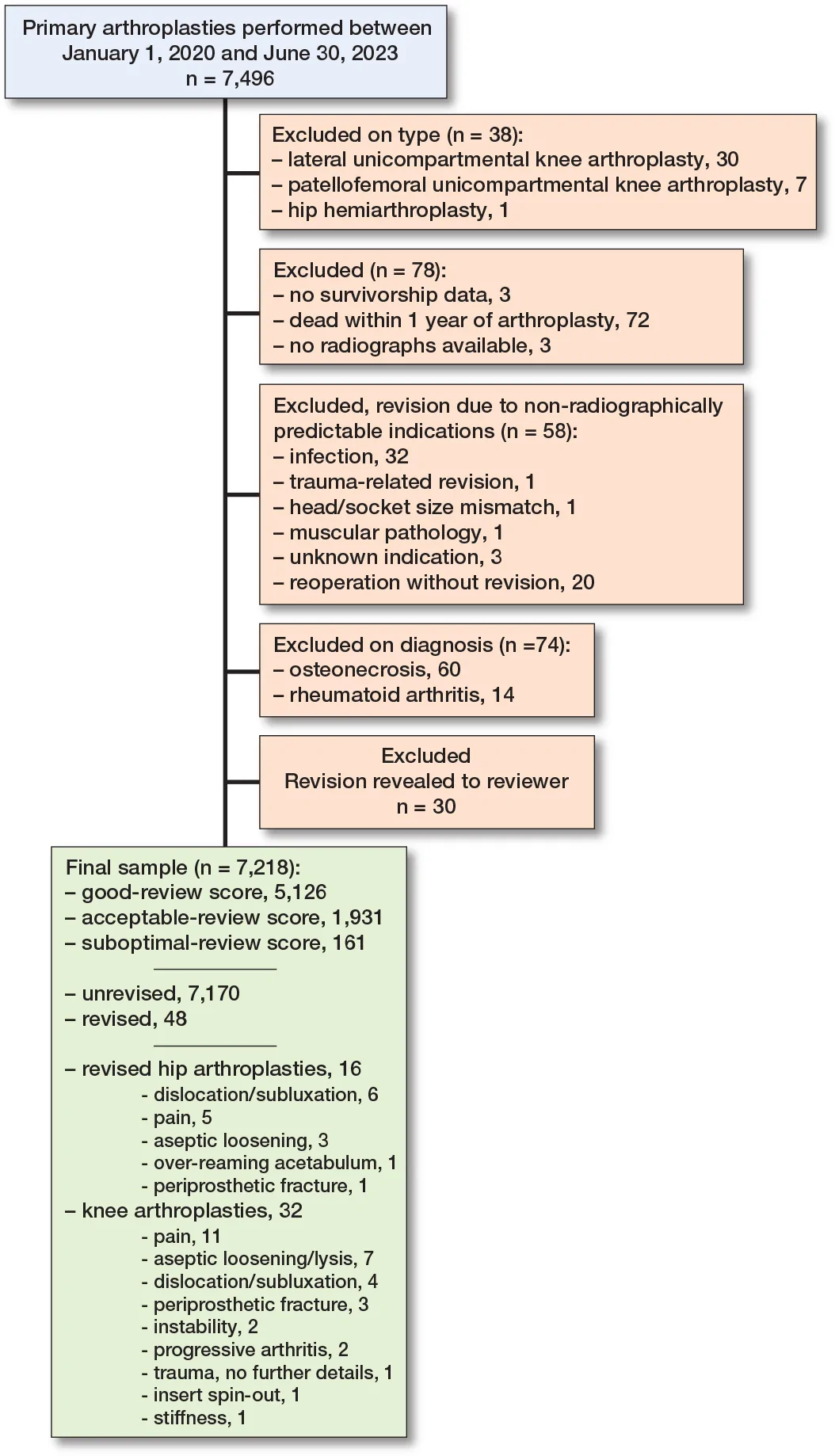
Flowchart showing which arthroplasties were excluded from the study.

Of the 7,496 arthroplasties recorded in the JointCare registry during the study window, 7,218 were included in the study sample, as shown in Figure 1. The cohort sizes were 5,126, 1,931, and 161 for the good-, acceptable-, and suboptimal-review-score cohorts, respectively (Table 2).

48 of the 7,218 arthroplasties underwent radiographically predictable aseptic revision, consisting of 24/5,126 (0.5%) in the good-review-score cohort, 16/1,931 (0.8%) in the acceptable-review-score cohort, and 8/161 (5.0%) in the suboptimal-review-score cohort. Indications for revisions are shown in Figure 1.

**Figure 2:**
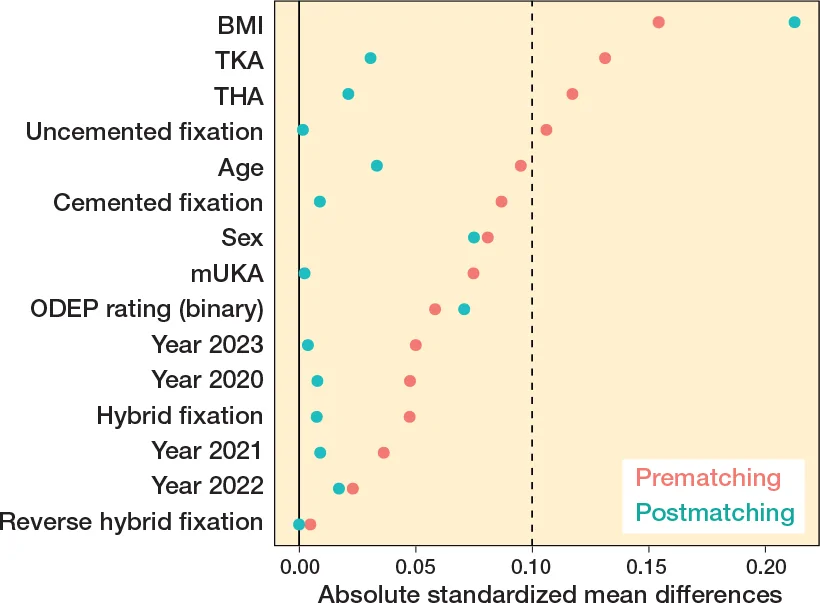
Love plot showing pre- and post-matching and weighting maximal absolute standardized mean differences between cohorts. For abbreviations, see Table 2.

The matching-and-weighting approach improved covariate balance across the review-score cohorts, reducing the ASMD from 0.07 to 0.03 for the overall sample, with all post-matching covariate imbalances, apart from BMI, below the threshold of 0.10. Pre- and post-matching ASMDs are shown in Figure 2, and post-matching cohort comparisons are given in Table 3. The ASMDs were reduced from 0.06 to 0.04 for total hip arthroplasties, 0.08 to 0.06 for total knee arthroplasties, and 0.14 to 0.12 for medial unicompartmental knee arthroplasties.

The post-matching effective sample sizes (ESSs) were 5,034, 1,687, and 112 for the good-, acceptable-, and suboptimal-review-score cohorts, respectively. These ESSs, although smaller than the original sample sizes (indicated in Table 2), were still sufficient to compare the cohorts. The substantially smaller ESS of the suboptimal cohort contributed to wider confidence intervals and less precise time-to-event estimates in that group.

### Outcome

**Figure 3:**
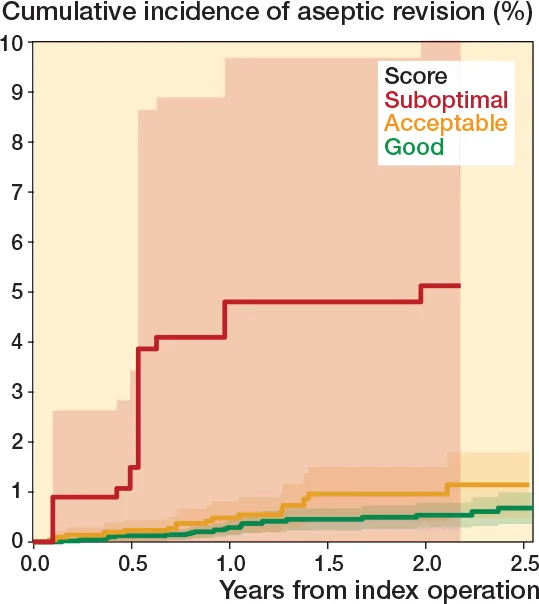
Weighted Kaplan–Meier curves for time to aseptic revision in the overall sample.

**Figure 4:**
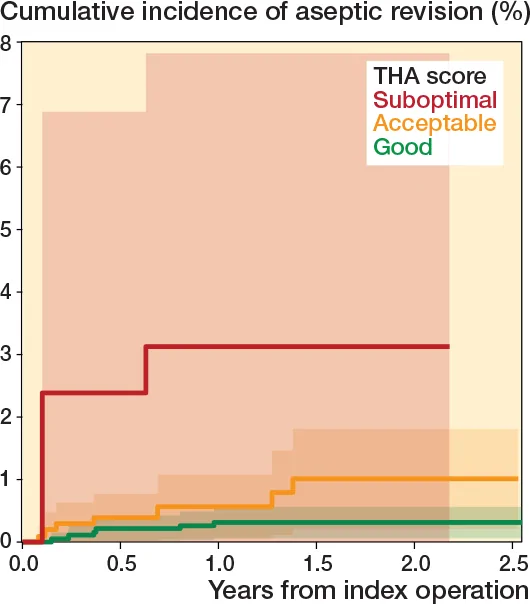
Weighted Kaplan–Meier curves for time to aseptic revision in total hip arthroplasties (THA).

**Figure 5:**
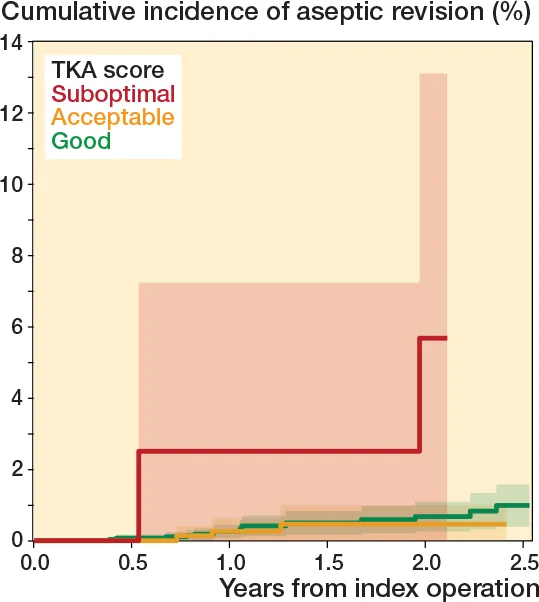
Weighted Kaplan–Meier curves for time to aseptic revision in total knee arthroplasties (TKA).

**Figure 6:**
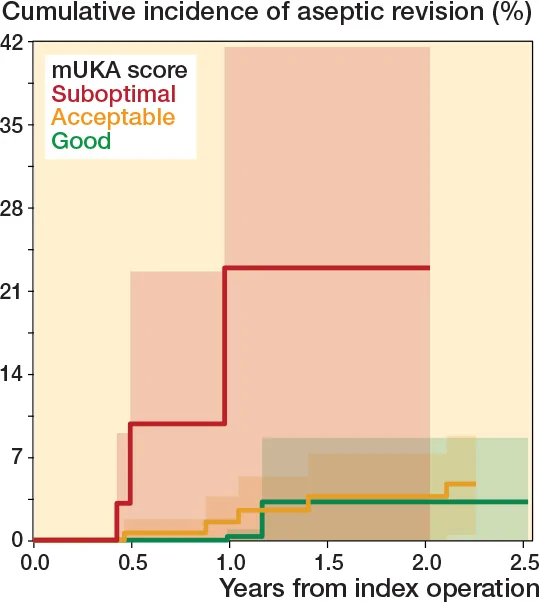
Weighted Kaplan–Meier curves for time to aseptic revision in medial unicompartmental knee arthroplasties (mUKA).

Arthroplasties in the suboptimal-review-score cohort had a significantly higher hazard ratio (HR) for aseptic revision, when compared with those in the good-review-score cohort (HR 9.4, CI 3.6–24.5; P < 0.001; Figure 3 and Table 4). Furthermore, arthroplasties in the suboptimal-review-score cohort had significantly higher HRs for aseptic revision, when compared with those in the good-review-score cohort, for total hip arthroplasties (HR 11.4, CI 2.6–49.7; P = 0.001; Figure 4 and Table 5), total knee arthroplasties (HR 5.7, CI 1.3–25.6; P = 0.02; Figure 5 and Table 6) and medial unicompartmental knee arthroplasties (HR 9.8, CI 2.0–47.4; P = 0.005; Figure 6 and Table 7). Proportional hazard assumptions were met for analyses of the overall sample, and for total hip and total knee arthroplasties. The event rate for medial unicompartmental knee arthroplasties was not sufficient to perform Schoenfeld residual tests. These associations should therefore be interpreted with caution, particularly in the procedure-specific subgroup analyses, because the number of events in the suboptimal cohort was small.

**Table 4:** Cox proportional hazards regression for the overall sample

Variable	Hazard ratio (CI)	P value
Suboptimal-review score ^**a**^	9.4 (3.6–24.5)	< 0.001
Acceptable-review score ^**a**^	1.6 (0.78–3.2)	0.2
BMI	1.1 (0.97–1.2)	0.2

a In relation to good-review score.

BMI = body mass index; CI = 95% confidence interval.

**Table 5:** Cox proportional hazards regression for total hip arthroplasties

Variable	Hazard ratio (CI)	P value
Suboptimal-review score ^**a**^	11 (2.6–49.7)	0.001
Acceptable-review score ^**a**^	2.9 (0.94–8.9)	0.06
ODEP-rated implant ^**b**^	0.51 (0.05–4.8)	0.6

a In relation to good-review score.

b In relation to non-ODEP-rated implants.

CI = 95% confidence interval; ODEP = Orthopaedic Data Evaluation Panel.

**Table 6:** Cox proportional hazards regression for total knee arthroplasties

Variable	Hazard ratio (CI)	P value
Suboptimal-review score ^**a**^	5.7 (1.3–26)	0.02
Acceptable-review score ^**a**^	0.58 (0.14–2.3)	0.4
BMI	1.0 (0.92–1.2)	0.5

a In relation to good-review score.

BMI = body mass index; CI = 95% confidence interval.

**Table 7:** Cox proportional hazards regression for medial unicompartmental knee arthroplasties

Variable	Hazard ratio (CI)	P value
Suboptimal-review score ^**a**^	9.8 (2.0–47)	0.005
Acceptable-review score ^**a**^	1.3 (0.25–6.4)	0.8
Age (years)	0.95 (0.90–1.0)	0.2
BMI	1.0 (0.93–1.2)	0.5
Uncemented implant fixation ^**b**^	0.50 (0.05–5.2)	0.6

Hybrid and reverse-hybrid implant fixation, and implant ODEP rating were included in the model, but results are omitted from this table because zero-event levels produced unstable coefficients.

a In relation to good-review score.

b In relation to cemented implant fixation.

BMI = body mass index; CI = 95% confidence interval; ODEP = Orthopaedic Data Evaluation Panel.

In the overall sample (HR 1.6, CI 0.78–3.2; P = 0.2; see Figure 3 and Table 4), and within each procedure (see Figures 4–6 and Tables 5–7), arthroplasties in the acceptable-review-score cohorts had higher hazards of aseptic revision than those in the good-review-score cohort; however, these results were not statistically significant.

## Discussion

To our knowledge, registry-based radiographic peer review is a unique contribution.

We aimed to assess the predictive value of peer review in identifying primary hip and knee arthroplasties at higher risk of aseptic revision. We found that arthroplasties given suboptimal-review scores were associated with a 9.4-fold increased risk of early aseptic revision compared with those with good-review scores. The benefit of a good- over an acceptable-review score is less clear, with no significant difference in revision rates observed between these cohorts. A contributing factor here is expected to be that the peer review program, used as a data source, prioritized suboptimal detection, while differentiating between good and acceptable scores was allocated substantially fewer resources.

The higher HR estimates for total hip and medial unicompartmental knee arthroplasties than for total knee arthroplasty may indicate that the revision risk of these procedures is more discernible via radiographic review. However, given the large CIs of the procedure subgroup analyses, and the lack of testing of procedure-by-review-score interactions or dependence of procedure-specific revision on radiographically evident factors, further research is needed before drawing such conclusions [13-17].

### Limitations

Several biases may be present in this study; however, they were not expected to meaningfully affect the results. Selection and information bias were deemed negligible given the low exclusion rate, as shown in Figure 1. The study is, however, limited to a portion of the private sector of South Africa. Recall and assessment biases were expected to be minimal, and confounders, while present, were addressed with the statistical methodology employed.

The setup of the peer review program is expected to influence accuracy, and the findings of this study may not be reproducible given other reviewers. The review program used in the study allocated sizable resources to performing accurate radiographic assessment. Great care was taken in reviewer selection, with reviewers themselves being assessed with respect to the overall reviewer group. Regular reviewer meetings were held so that cases, and acceptable levels of care, could be discussed. As such, the costs resulting from running a peer review program are not insubstantial.

The suboptimal cohort was much smaller than the other cohorts, with fewer events. As a result, the HR confidence intervals were wide and the weighted Kaplan–Meier curves, especially in the later follow-up period and in procedure-specific subgroup analyses, should be interpreted as indicating the direction of association rather than a precisely estimated magnitude of risk stratification.

The additional expense of the peer review program used in this study is estimated to be between 2% and 4% of each primary arthroplasty’s total cost. The additional cost estimate is complicated by various factors, including economies of scale, the integration of the financial and peer review systems, and the savings that result from the billing errors the peer review program identifies. A detailed analysis of the value of running a radiographic peer review program is recommended.

### Conclusion

Radiographic review by experienced arthroplasty surgeons was able to identify arthroplasties at higher risk of aseptic revision.

Therefore, radiographic peer review may prove to be a vital tool for value-based care. As a measure of quality, radiographic peer review could be used to help identify areas with significant room for improvement, thereby improving future care. Registries should therefore consider capturing peer-review or radiographic-quality variables, rather than relying on revision endpoints alone.

### Supplementary data

The data analysis code is available as supplementary data on the article homepage, doi: 10.2340/17453674.2026.46431

ASDD was the primary author, and contributed to protocol development, preliminary statistical analysis, manuscript drafting, and proofreading. IDL and PF contributed to protocol development, manuscript drafting, and proofreading. AD contributed to statistical analysis, generation of tables and figures, manuscript drafting, and proofreading.

## Supplementary Material



## Notes

Handling co-editors: Keijo Mäkelä and Philippe Wagner

*Acta* thanks Eduardo García-Rey and Rihard Trebse for help with peer review of this manuscript.
