# Supplementary material for: The accuracy of radiographic peer review in identifying primary hip and knee arthroplasties at higher risk of early aseptic revision: an observational cohort study from the JointCare registry of South Africa
Source: Acta Orthop. 2026 Sep 3;97:587–94. doi: 10.2340/17453674.2026.46431 (PMC13543271; doi:10.2340/17453674.2026.46431)
Supplement: Supplementary file 1 [file ActaO-97-46431-s1.pdf]

```
#R version 4.5.1

#Import packages
library(cobalt) #version 4.6.1
library(MatchIt) #version 4.7.2
library(quickmatch) #version 0.2.3
library(readxl) #version 1.5.0
library(survey) #version 4.4.8
library(survival) #version 3.8.3
library(survminer) #version 0.5.1

#Import data
df <- read_excel("dataset.xlsx", guess_max = Inf)

#Use reference-invariate contrasts for propensity score matching (PSM)
options(contrasts = c("contr.sum", "contr.poly"))

#Define independent variables
for (num_var in c("age", "BMI")){
  df[[num_var]] <- as.numeric(df[[num_var]])
}

for (unordered_var in c("sex", "procedure", "ODEP_rated", "implant_fixation",
"review_score")){
  df[[unordered_var]] <- factor(df[[unordered_var]], ordered = FALSE)
}
df$review_score <- relevel(df$review_score, ref = "Good")

df$year <- factor(df$year, ordered = TRUE)

#Binarise outcome variable
df$revision <- as.numeric(df$revision)

#Create matrix of independent variables
df_matrix <- model.matrix(~ age + sex + BMI + year + procedure + ODEP_rated +
implant_fixation, data = df)[, -1, drop = FALSE]

#Calculate robust Mahalanobis distances
dist_matrix <- robust_mahalanobis_dist(data = as.data.frame(df_matrix))

#Apply generalised full PSM
PSM <- quickmatch(distances = dist_matrix, treatments = df$review_score)
PSM_numeric <- as.numeric(PSM)

#Extract weights
PSM_weights <- as.numeric(matching_weights(df$review_score, PSM))

#Assess balance and get list of poorly matched variables to add to doubly robust Cox model
##with balance table
bal <- bal.tab(
  review_score ~ age + sex + BMI + year + procedure + ODEP_rated + implant_fixation,
  data = df,
  weights = PSM_weights,
  method   = "weighting",
  estimand = "ATE",
  s.d.denom = "pooled",
  un = TRUE
  )
bal

collapse_to_existing <- function(vars, data) {
  out <- vars
  for (i in seq_along(out)) {
    v <- out[i]
    if (v %in% names(data)) next
    base <- sub("_[^_]+$", "", v)
    if (base %in% names(data)) out[i] <- base
  }
  unique(out)
```

```
}

imbalanced_raw <- rownames(bal$Balance)[bal$Balance$Max.Diff.Adj > 0.1]
imbalanced_vars <- collapse_to_existing(imbalanced_raw, df)

##with Love plot
love.plot(
  x = ~ age + sex + BMI + year + procedure + ODEP_rated + implant_fixation,
  treat = df$review_score,
  data = df,
  weights = PSM_weights,
  estimand = "ATE",
  abs = TRUE,
  thresholds = c(m = 0.10),
  var.order = "unadjusted",
)

##with pre- and post-matching cohort comparisons
design <- svydesign(ids = ~1, data = df, weights = ~PSM_weights)

###for numerical variables
for (num_var in c("age", "BMI")){
  print(num_var)
  print("Pre-matching")
  print(tapply(df[[num_var]], df$review_score, quantile, probs = c(0.25, 0.5, 0.75), na.rm
= TRUE))
  print(kruskal.test(df[[num_var]] ~ df$review_score))
  print("Post-matching")
  print(svyby(as.formula(paste0("~", num_var)), ~ review_score, design, svyquantile,
c(0.25, 0.5, 0.75)))
  print(svyranktest(reformulate("review_score", response = num_var), design, test =
"KruskalWallis"))
}

###for categorical variables
for (cat_var in c("sex", "ASA", "year", "procedure", "ODEP_rated", "implant_fixation")){
  print(cat_var)
  print("Pre-matching")
  print(round(prop.table(table(df[[cat_var]], df$review_score), margin = 2) * 100, digits
= 1))
  print(chisq.test(df[[cat_var]], df$review_score))
  print("Post-matching")
  print(round(prop.table(svytable(as.formula(paste0("~", cat_var, "+ review_score")),
design), margin = 2) *100, digits = 1))
  print(svychisq(as.formula(paste0("~", cat_var, "+ review_score")), design, statistic =
"F"))
}

###Make list of variables differing significantly among cohorts
sig_vars <- c()

for (num_var in c("age", "BMI")){
  post_matching_p <- svyranktest(reformulate("review_score", response = num_var), design,
test = "KruskalWallis")$p.value
  if (!is.na(post_matching_p) && post_matching_p < 0.05){
    sig_vars <- c(sig_vars, num_var)
  }
}

for (cat_var in c("sex", "ASA", "year", "procedure", "ODEP_rated", "implant_fixation")){
  post_matching_p <- suppressWarnings(svychisq(as.formula(paste0("~", cat_var, "+
review_score")), design, statistic = "F")$p.value)
  if (!is.na(post_matching_p) && post_matching_p < 0.05){
    sig_vars <- c(sig_vars, cat_var)
  }
}

sig_vars <- unique(sig_vars)
```

```
#Make list of variables to add to doubly robust Cox model
vars_to_add <- unique(c(imbalanced_vars, sig_vars))

#Set reference values for Cox regression
contrasts(df$review_score) <- contr.treatment(length(levels(df$review_score)), base =
which(levels(df$review_score) == "Good"))
contrasts(df$sex) <- contr.treatment(length(levels(df$sex)), base = which(levels(df$sex)
== "male"))
contrasts(df$year) <- contr.treatment(length(levels(df$year)), base =
which(levels(df$year) == "2022"))
contrasts(df$ODEP_rated) <- contr.treatment(length(levels(df$ODEP_rated)), base =
which(levels(df$ODEP_rated) == 0))
contrasts(df$implant_fixation) <- contr.treatment(length(levels(df$implant_fixation)),
base = which(levels(df$implant_fixation) == "cemented"))

fit_PSM <- coxph(as.formula(paste("Surv(survivorship_days, revision) ~",
paste(c("review_score", vars_to_add, "cluster(PSM_numeric)"), collapse = " + "))),
                 data = df,
                 weights = PSM_weights,
                 ties = "efron",
                 robust = TRUE)
print(summary(fit_PSM))

#Assess proportional hazards assumptions
zph <- cox.zph(fit_PSM, transform = "km")
print(zph, digits = 3)

#Clean up
rm(list = ls())

#Subset analyses for each procedure type
for (proc in c("THA", "TKA", "mUKA")){
  df <- read_excel("dataset.xlsx", guess_max = Inf)
  print(proc)

  #Create subset of dataframe
  df_subset <- subset(df, df$procedure == proc)

  #Use reference-invariate contrasts for propensity score matching (PSM)
  options(contrasts = c("contr.sum", "contr.poly"))

  #Define independent variables
  for (num_var in c("age", "BMI")){
    df_subset[[num_var]] <- as.numeric(df_subset[[num_var]])
  }

  for (unordered_var in c("sex", "ODEP_rated", "implant_fixation", "review_score")){
    df_subset[[unordered_var]] <- factor(df_subset[[unordered_var]], ordered = FALSE)
  }
  df_subset$review_score <- relevel(df_subset$review_score, ref = "Good")

  df_subset$year <- factor(df_subset$year, ordered = TRUE)

  #Binarise outcome variable
  df_subset$revision <- as.numeric(df_subset$revision)

  #Create matrix of independent variables
  df_matrix <- model.matrix(~ age + sex + BMI + year + ODEP_rated + implant_fixation, data
= df_subset)[, -1, drop = FALSE]

  #Calculate robust Mahalanobis distances
  dist_matrix <- robust_mahalanobis_dist(data = as.data.frame(df_matrix))

  #Apply generalised full PSM
  PSM <- quickmatch(distances = dist_matrix, treatments = df_subset$review_score)
  PSM_numeric <- as.numeric(PSM)

  #Extract weights
  PSM_weights <- as.numeric(matching_weights(df_subset$review_score, PSM))
```

```
  #Get list of poorly matched variables to add to doubly robust Cox model
  ##with balance table
  bal <- bal.tab(
    review_score ~ age + sex + BMI + year + ODEP_rated + implant_fixation,
    data = df_subset,
    weights = PSM_weights,
    method   = "weighting",
    estimand = "ATE",
    s.d.denom = "pooled",
    un = TRUE
  )

  collapse_to_existing <- function(vars, data) {
    out <- vars
    for (i in seq_along(out)) {
      v <- out[i]
      if (v %in% names(data)) next
      base <- sub("_[^_]+$", "", v)
      if (base %in% names(data)) out[i] <- base
    }
    unique(out)
  }

  imbalanced_raw <- rownames(bal$Balance)[bal$Balance$Max.Diff.Adj > 0.1]
  imbalanced_vars <- collapse_to_existing(imbalanced_raw, df_subset)

  ##with pre- and post-matching cohort comparisons
  design <- svydesign(ids = ~1, data = df_subset, weights = ~PSM_weights)
  sig_vars <- c()

  for (num_var in c("age", "BMI")){
    post_matching_p <- svyranktest(reformulate("review_score", response = num_var),
design, test = "KruskalWallis")$p.value
    if (!is.na(post_matching_p) && post_matching_p < 0.05){
      sig_vars <- c(sig_vars, num_var)
    }
  }

  for (cat_var in c("sex", "ASA", "year", "ODEP_rated", "implant_fixation")){
    post_matching_p <- suppressWarnings(svychisq(as.formula(paste0("~", cat_var, "+
review_score")), design, statistic = "F")$p.value)
    if (!is.na(post_matching_p) && post_matching_p < 0.05){
      sig_vars <- c(sig_vars, cat_var)
    }
  }

  sig_vars <- unique(sig_vars)

  #Make list of variables to add to doubly robust Cox model
  vars_to_add <- unique(c(imbalanced_vars, sig_vars))

  #Set reference values for Cox regression
  contrasts(df_subset$review_score) <-
contr.treatment(length(levels(df_subset$review_score)), base =
which(levels(df_subset$review_score) == "Good"))
  contrasts(df_subset$sex) <- contr.treatment(length(levels(df_subset$sex)), base =
which(levels(df_subset$sex) == "male"))
  contrasts(df_subset$year) <- contr.treatment(length(levels(df_subset$year)), base =
which(levels(df_subset$year) == "2022"))
  contrasts(df_subset$ODEP_rated) <- contr.treatment(length(levels(df_subset$ODEP_rated)),
base = which(levels(df_subset$ODEP_rated) == 0))
  contrasts(df_subset$implant_fixation) <-
contr.treatment(length(levels(df_subset$implant_fixation)), base =
which(levels(df_subset$implant_fixation) == "cemented"))

  fit_PSM <- coxph(as.formula(paste("Surv(survivorship_days, revision) ~",
paste(c("review_score", vars_to_add, "cluster(PSM_numeric)"), collapse = " + "))),
                   data = df_subset,
```

```
                  weights = PSM_weights,
                  ties = "efron",
                  robust = TRUE)
  print(summary(fit_PSM))

  #Assess proportional hazards assumptions
  zph <- tryCatch(
    cox.zph(fit_PSM, transform = "km"),
      error = function(e) {
        "Error calculating Schoenfeld residuals"
    }
  )
  print(zph, digits = 3)

  #Clean up
  rm(list = ls())
}
```
